# Evaluation of machine learning algorithms for predicting direct-acting antiviral treatment failure among patients with chronic hepatitis C infection

**DOI:** 10.1038/s41598-022-22819-4

**Published:** 2022-10-27

**Authors:** Haesuk Park, Wei-Hsuan Lo-Ciganic, James Huang, Yonghui Wu, Linda Henry, Joy Peter, Mark Sulkowski, David R. Nelson

**Affiliations:** 1 Department of Pharmaceutical Outcomes and Policy, College of Pharmacy, University of Florida, HPNP Building Room 3325, 1225 Center Drive, Gainesville, FL 32610 USA; 2 Health Outcomes & Biomedical Informatics, College of Medicine, University of Florida, Gainesville, FL USA; 3 Department of Medicine, University of Florida, Gainesville, FL USA; 4 Johns Hopkins University School of Medicine, Baltimore, MD USA

**Keywords:** Gastroenterology, Medical research

## Abstract

Despite the availability of efficacious direct-acting antiviral (DAA) therapy, the number of people infected with hepatitis C virus (HCV) continues to rise, and HCV remains a leading cause of liver-related morbidity, liver transplantation, and mortality. We developed and validated machine learning (ML) algorithms to predict DAA treatment failure. Using the HCV-TARGET registry of adults who initiated all-oral DAA treatment, we developed elastic net (EN), random forest (RF), gradient boosting machine (GBM), and feedforward neural network (FNN) ML algorithms. Model performances were compared with multivariable logistic regression (MLR) by assessing C statistics and other prediction evaluation metrics. Among 6525 HCV-infected adults, 308 patients (4.7%) experienced DAA treatment failure. ML models performed similarly in predicting DAA treatment failure (C statistic [95% CI]: EN, 0.74 [0.69–0.79]; RF, 0.74 [0.69–0.80]; GBM, 0.72 [0.67–0.78]; FNN, 0.75 [0.70–0.80]), and all 4 outperformed MLR (C statistic [95% CI]: 0.51 [0.46–0.57]), and EN used the fewest predictors (n = 27). With Youden index, the EN had 58.4% sensitivity and 77.8% specificity, and nine patients were needed to evaluate to identify 1 DAA treatment failure. Over 60% treatment failure were classified in top three risk decile subgroups. EN-identified predictors included male sex, treatment < 8 weeks, treatment discontinuation due to adverse events, albumin level < 3.5 g/dL, total bilirubin level > 1.2 g/dL, advanced liver disease, and use of tobacco, alcohol, or vitamins. Addressing modifiable factors of DAA treatment failure may reduce the burden of retreatment. Machine learning algorithms have the potential to inform public health policies regarding curative treatment of HCV.

## Introduction

Hepatitis C virus (HCV) is the most common chronic bloodborne infection in the U.S. and a leading cause of liver-related morbidity, liver transplantation, and mortality^1–4^. The number of deaths resulting from HCV has exceeded the total number of deaths due to 60 other infectious diseases combined, including HIV, pneumococcal disease, and tuberculosis, and the rate of HCV infection continues to increase^1,5^. In 2014, HCV treatment greatly improved with the approval of direct-acting antiviral (DAA) therapy, with therapeutic efficacy of 95% or higher^6^, yet new cases of this preventable disease are still increasing^7–12^. With less than a decade remaining to reach the goal set by the World Health Organization to eliminate viral hepatitis by reducing new hepatitis infections by 90% and to reduce death due to hepatitis infections by 65% by 2030^13^, HCV remains one of the top causes of chronic liver disease worldwide^14^.

The rate of treatment failures, though small in percentage (< 5%), remains large in number considering that the magnitude of patients with HCV means that a substantial number of first DAA therapy interventions will fail and that more patients will need to be treated in the near future^15–17^. To understand more about patients who are at high risk of treatment failure, a recent study by members of our team used machine learning to identify pretreatment risk factors to help clinicians identify factors that may be modifiable before initiation of DAA treatment^18^. That study found that an algorithm using a gradient boosting machine (GBM) performed effectively for DAA treatment risk prediction with 41 pretreatment predictors; the top 10 GBM-identified predictors included albumin, liver enzyme, and total bilirubin levels, sex, HCV viral load, sodium level, presence of hepatocellular carcinoma, platelet level and tobacco use^18^.

However, despite the best performance by the GBM algorithm among the four machine learning algorithms assessed in that study, the C statistic was suboptimal (0.69), suggesting that there may be important predictors not included in the model. We hypothesized here that predictors present during treatment, such as the duration of treatment, adherence to the prescribe medication treatment, and adverse events experienced, may better explain treatment failure when combined with patient characteristics present prior to treatment. Therefore, we developed and validated additional machine learning algorithms to predict DAA treatment failure, this time using predictors both before and during treatment. An overarching goal of the present study was to provide clinicians with a reasonable list of risk factors that may be amendable to modification prior to or during treatment of HCV to reduce the rate of DAA treatment failure.

## Methods

### Study design and participants

The data for this cohort study were obtained from HCV-TARGET (Hepatitis C Therapeutic Registry and Research Network), a longitudinal, prospective, observational cohort study of the real-world administration of DAA therapy^19^. The use by HCV-TARGET of a standardized and centralized method to abstract data from medical records combined with a detailed data monitoring system ensures both the quality and integrity of the database. Patients included in the present study were 18 years of age or older, initiated an all-oral DAA HCV treatment regimen between February 2014 and 2018, had virologic outcome data available as of June 2019, and provided written informed consent to participate in HCV-TARGET. The DAA regimens used with or without ribavirin included ledipasvir/sofosbuvir; sofosbuvir plus daclatasvir; ombitasvir/paritaprevir/ritonavir plus dasabuvir; elbasvir/grazoprevir; sofosbuvir/velpatasvir; sofosbuvir/velpatasvir/voxilaprevir; and glecaprevir/pibrentasvir. Patients who completed the assigned HCV treatment or who discontinued treatment early owing to efficacy concerns or adverse effects and who had virologic outcome data available were assigned to the per-protocol population. This report complies with the Transparent Reporting of a Multivariable Prediction Model for Individual Prognosis or Diagnosis (TRIPOD) guideline (Supplementary Table 1)^20^. This study was approved by the institutional review board of the University of Florida, Gainesville.

### Primary outcome

Sustained virologic response (SVR) was the primary efficacy end point and was defined as plasma HCV RNA levels undetectable or below quantitation 12 or more weeks after completing DAA treatment.

### Predictor candidates

Given the results in prior studies^21–23^ as well as previous work by members of our team^18^, we assessed 359 variables as candidate predictors. The candidates included sociodemographic and clinical characteristics, DAA treatment regimen and duration, and laboratory test results consisting of 242 predictors before therapy initiation and 117 predictors 4 weeks after initiation (Supplementary Table 2).

### Approaches to machine learning and evaluation of prediction model performance

A full description of the machine learning approach used in the present study was previously described^18^. In brief, our analyses generated risk prediction scores for DAA treatment failure per individual and then used these risk prediction scores to group individuals into subgroups comprising similar DAA treatment failure risks. Two-thirds of patients were randomly assigned to a training sample for development of a prediction algorithm, which was then validated with the remaining one-third of patients. The prediction results of four commonly used machine learning approaches shown to have the best prediction results, elastic net (EN), random forest (RF), GBM, and feedforward neural network (FNN)^24,25^, were compared with a multivariable logistic regression algorithm previously generated from HCV-TARGET data, which included as predictors male sex, albumin level, platelet count, total bilirubin level, history of previous treatment, proton pump inhibitor [PPI] use, and HCV genotype subtype^26^. The Methods in the Supplement describe the details for each of the machine learning approaches used. We assessed the discrimination performance ability of the various models by comparing the DAA treatment failure rates of patients who had been predicted to be high risk of DAA treatment failure vs. the patients who had been predicted to be at low risk. Using the DeLong test, the C statistics (0.7–0.8 considered good; > 0.8, very good) and precision-recall curves for the various models were compared using the validation sample^27^. Eight metrics of evaluation were also assessed: sensitivity, specificity, positive predictive value (PPV), negative predictive value (NPV), positive likelihood ratio, negative likelihood ratio, number needed to evaluate (NNE) to identify 1 DAA treatment failure, and estimated positive alert rate. For the EN final model, we report beta coefficients and odds ratios. Because EN regularization does not provide an estimate of precision, 95% CIs are not given herein.

Supplementary Table 3 gives the prediction metrics at numerous sensitivity and specificity levels, including the arbitrary selection of 90% sensitivity as an anchor and the use of the Youden index to obtained a threshold with balanced sensitivity and specificity^28^. To enable a more granular examination of patients at highest risk of DAA treatment failure, the validation sample was split into subgroups using the decile of the predicted risk score from the training algorithm. The highest decile was further split into three strata: the top first percentile, the second through fifth percentile, and the sixth through tenth percentile. Calibration plots were used to compare the agreement between the observed and predicted risk of DAA treatment failure for each risk subgroup. For potential clinical use, we report the top 25 most important prediction factors. For comparison of the training and validation samples, patient characteristics were analyzed using 2-tailed *t* tests for continuous variables and chi-square tests for categorical variables. All analyses were performed using SAS, version 9.4 (SAS Institute Inc, Cary, North Carolina) and Python, version 3.6 (Python Software Foundation, Delaware).

## Results

### Patient characteristics

In total, 6525 patients (4894 in the training sample and 1631 in the validation sample) were included in this study. The sociodemographic (e.g. mean [SD] age, 57 [11] years; 60% male; 23% Black race and ethnicity; and 66% White race and ethnicity) and clinical characteristics (e.g. 36% with cirrhosis) of the training and validation groups were similar (Table 1**)**. Approximately 50% of participants had HCV genotype subtype 1a, 15% had a viral load of at least 6 million IU/mL, 14% had a history of hepatic decompensation, and 8% had undergone a prior liver transplantation. Overall, 65% were treatment naïve and the three most commonly used DAA treatment regimens for the remaining patients were ledipasvir/sofosbuvir (39%), followed by sofosbuvir/velpatasvir (11%), and then glecaprevir/pibrentasvir (9%). At baseline, approximately 28% of patients reported PPI use, 35% reported vitamin use, 60% reported tobacco product use; and 29% reported alcohol use. Overall, 6217 patients (95.3%) achieved an SVR; however, 308 patients (4.7%) experienced DAA treatment failure.

**Table 1:** Baseline sociodemographic and clinical characteristics of patients treated with all-oral DAAs and DAA treatment failure rates, stratified by training and validation samples.

Characteristic	No. (%) of patients in sample	
	Training (n = 4894)	Validation (n = 1631)
Age, mean (SD), y	57.51 (10.6)	57.50 (10.5)
**Sex**		
Male	2978 (60.9)	982 (60.2)
**Race and ethnicity**		
White	3259 (66.6)	1080 (66.2)
Black	1126 (23.0)	378 (23.2)
Other/not reported	509 (10.4)	173 (10.6)
Cirrhosis	1780 (36.4)	564 (34.6)
Decompensated cirrhosis	688 (14.1)	225 (13.8)
Hepatocellular carcinoma	295 (6.0)	84 (5.2)
Liver transplantation	371 (7.6)	124 (7.6)
**HCV genotype**		
1a	2,404 (49.1)	773 (47.4)
1b	1,162 (23.7)	383 (23.5)
3	650 (13.2)	204 (12.5)
Other/unknown	678 (13.9)	271 (16.6)
HCV RNA ≥ 6 million IU/mL	739 (15.1)	250 (15.3)
Albumin level ≥ 3.5 g/dL	3,784 (77.3)	1,252 (76.8)
Total bilirubin level ≤ 1.2 g/dL	4,268 (87.2)	1,433 (87.9)
Platelet count, median (range), × 10^3^/μL	182 (6–746)	182 (3–548)
MELD score, median (range)	8 (6–39)	8 (6–33)
**DAAs**		
Ledipasvir/sofosbuvir	1916 (39.1)	624 (38.2)
Ledipasvir/sofosbuvir + RBV	314 (6.4)	103 (6.4)
Ombitasvir/paritaprevir/ritonavir + dasabuvir	214 (4.4)	87 (5.3)
Ombitasvir/paritaprevir/ritonavir + dasabuvir + RBV	423 (8.6)	143 (8.8)
Glecaprevir/pibrentasvir	459 (9.4)	152 (9.3)
Glecaprevir/pibrentasvir + RBV	1 (0.0)	1 (0.0)
Elbasvir/grazoprevir	375 (7.7)	127 (7.8)
Elbasvir/grazoprevir + RBV	23 (0.5)	9 (0.5)
Sofosbuvir + daclatasvir	236 (4.8)	72 (4.4)
Sofosbuvir + daclatasvir + RBV	169 (3.5)	55 (3.4)
Sofosbuvir/velpatasvir	562 (11.5)	185 (11.3)
Sofosbuvir/velpatasvir + RBV	125 (2.5)	53 (3.3)
Sofosbuvir/velpatasvir/voxilaprevir	77 (1.6)	20 (1.2)
**Treatment status**		
Naïve	3139 (64.1)	1068 (65.5)
Experienced	1309 (26.7)	434 (26.6)
DAA-experienced	284 (5.8)	86 (5.3)
**Baseline use**		
Proton pump inhibitor	1350 (27.6)	465 (28.5)
Tobacco	2910 (59.5)	1011 (62.0)
Alcohol	1362 (27.8)	539 (33.0)
Vitamins	1715 (35.0)	552 (33.8)

*DAA* direct-acting antiviral, *HCV* hepatitis C virus, *MELD* model for end-stage liver disease, *RBV* ribavirin.

### Prediction performance

**Figure 1:**
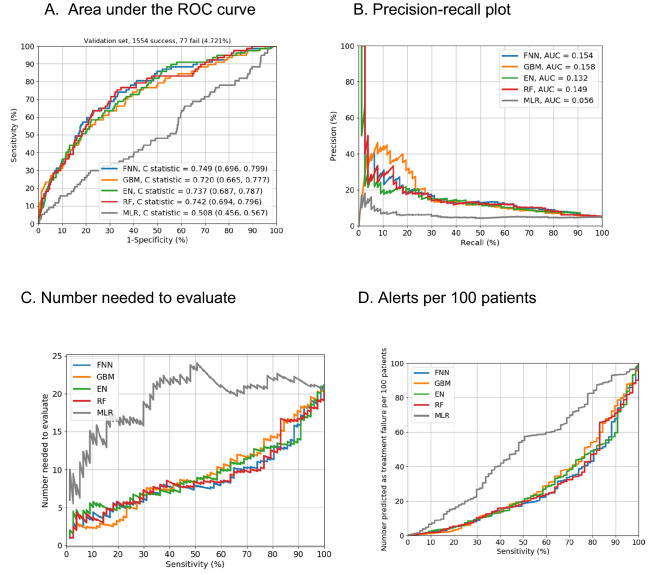
Prediction performance of 4 machine learning models vs. a multivariable logistic regression model. *FNN* feedforward neural network, *EN* elastic net, *GBM* gradient boosting machine, *MLR* multivariable logistic regression, *RF* random forest, *ROC* receiver operating characteristic. Data were derived using the validation sample, which comprised 1631 patients: 1554 with direct-acting antiviral treatment success, and 77 with treatment failure. (**A**), Results include C statistics and 95% CIs. (**B**), precision is equivalent to the positive predictive value, and recall to sensitivity). Models with curves closer to the upper right corner or above another curve have improved performance. (**C**), Number needed to evaluate by different cutoff points for sensitivity. (**D**), Alerts per 100 patients by various sensitivity cutoff points.

The ability to predict DAA treatment failure was similar across the four machine learning models, which all performed better than the previously developed multivariable logistic regression model: EN (C statistic = 0.74, 95% CI = 0.69–0.79), FNN (C statistic = 0.75, 95% CI = 0.70–0.80), RF (C statistic = 0.74, 95% CI = 0.69–0.80), GBM (C statistic = 0.72, 95% CI = 0.67–0.78), and multivariable logistic regression (C statistic = 0.51, 95% CI = 0.46–0.57) (Fig. 1A). Of the four machine learning models, the EN required the fewest predictors (EN = 27 vs. GBM = 47, FNN = 359 and RF = 268), and the GBM had slightly better precision-recall performance (Fig. 1B) as assessed by the area under the receiving operating characteristic curves. Supplementary Table 3 presents the prediction performance measures by sensitivity and specificity levels (90–100%). With performance measures balanced for sensitivity and specificity (using the Youden index), the EN approach had 58.4% sensitivity, 77.8% specificity, 11.5% PPV, 97.4% NPV, 9 needed to identify 1 DAA treatment failure, and 24 positive alerts per 100 patients. After setting the sensitivity to 90% to attempt to identify 90% of the observed DAA treatment failure, the EN had 44.3% specificity, 7.4% PPV, 98.9% NPV, 14 needed to identify 1 DAA treatment failure, and 57 positive alerts per 100 patients (Fig. 1C,D; Supplementary Table 3).

### Risk stratification by decile risk subgroup

**Figure 2:**
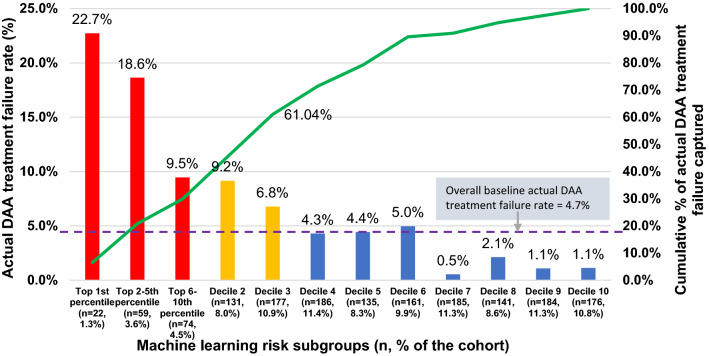
Electric net model assessment of direct-acting antiviral (DAA) treatment failure rates for each decile risk subgroup. Data were derived using the validation sample.

**Figure 3:**
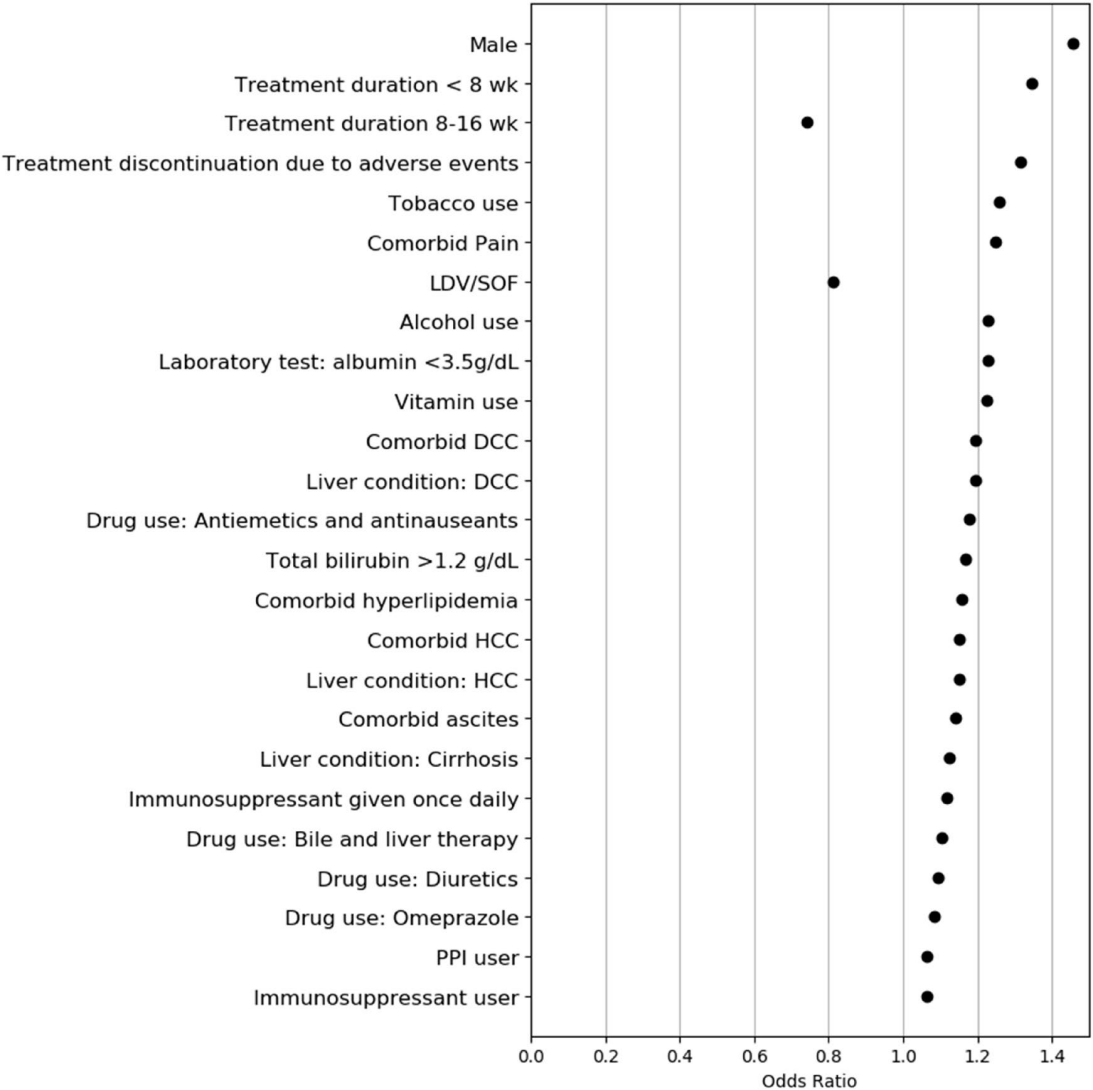
Elastic net model identification of 25 key prediction factors associated with direct-acting antiviral treatment failure^a^. *DCC* decompensated cirrhosis, *HCC* hepatocellular carcinoma, *LDV/SOF* ledipasvir/sofosbuvir, *PPI* proton pump inhibitor. ^a^Predictors are ordered by factor importance based on odds ratios. Elastic net regularization does not provide an estimate of precision; therefore, 95% CIs are not given.

The observed (actual) DAA treatment failure rates for patients by each decile subgroup using the EN model are shown in Fig. 2. The high-risk subgroup (with risk scores in the top decile; 155 patients [9.5%] in the validation sample) had a PPV of 14.8% and needed 7 to identify 1 treatment failure. Of 77 patients with DAA treatment failure, 47 (61%) were assigned to 1 of the top 3 subgroups (decile 1 = 30%, decile 2 = 15.6%, and decile 3 = 15.6%), that is, 7–22 per 100 patients. The DAA treatment failure rate in the first decile subgroup was at least 20-fold as high as that in the lower-risk groups (e.g. first percentile = 22.7% vs. tenth decile = 1.1%). Minimal rates of DAA treatment failure were found in the fourth through tenth decile subgroups (0.5–5 per 100 patients). The top 25 key predictors as identified by the EN model are shown in Fig. 3. Those predictors included male sex, DAA treatment duration < 8 weeks, treatment discontinuation due to adverse events, advanced liver disease (i.e. cirrhosis and hepatocellular carcinoma), albumin level < 3.5 g/dL, total bilirubin level > 1.2 g/dL, alcohol or tobacco use, pain, hyperlipidemia, and use of immunosuppressants, PPI, or vitamins.

## Discussion

In this cohort study, we used HCV-TARGET registry data to develop and evaluate four machine learning models to predict DAA treatment failure among patients treated for HCV. All four models showed good discrimination performance, outperforming models developed and assessed in a previous study by members of our group^18^ (C statistics 0.72–0.75 vs. 0.64–0.69) and substantially outperforming the multivariable logistic regression model result (C statistic, 0.51)^29^. As expected, the PPV was low given the low incidence of DAA treatment failure. However, the EN algorithm effectively subdivided the sample into different risk groups based on predicted risk scores, with 60% of the individuals with DAA treatment failure placed in the top three decile risk subgroups. The EN model was also the preferred and most parsimonious algorithm because it required only 25 predictors (vs. 359 predictors for FNN), reducing computational time. Identifying these risk subgroups as well as risk factors may inform treatment strategies for patients with HCV.

In the previous machine learning study by members of our group using the same dataset as in the present study (HCV-TARGET), only predictor candidates of DAA treatment failure before DAA treatment were assessed. That study found that the factors most strongly associated with treatment failure were albumin, bilirubin and liver enzyme (aspartate aminotransferase, alkaline phosphatase) levels, male sex, HCV RNA level, and the presence of advanced liver disease^18^. In the present study, we assessed the same pretreatment factors but also included factors associated with HCV treatment. Although low albumin levels, high bilirubin levels, male sex, and presence of advanced liver disease remained significant as predictors of DAA treatment failure, the other two factors (e.g. liver enzymes, HCV RNA level) previously found to be significant predictors did not. The most significant prediction factors in the present study were male sex, treatment duration < 8 weeks, or treatment discontinuation due to adverse effects, suggesting that treatment continuation is a key factor for achieving SVR. Although we do not know why patients discontinued early, further analysis indicated that 44% of patients had treatment durations of < 8 weeks and discontinued treatment due to adverse effects (data not shown). Of those, 38% had ribavirin, which is known to be associated with anemia, nausea, pulmonary, and dermatologic adverse conditions^30^.

The use of machine learning statistical approaches enabled us to confirm the results of prior studies that found similar baseline predictors of treatment failure, including factors associated with advanced liver disease or with treatment for advanced liver disease (e.g. use of immunosuppressants, diuretics, pain medication, or bile and liver therapies), PPI use, albumin level < 3.5 g/dL, total bilirubin level > 1.2 g/dL, and male sex, and predictors associated with treatment discontinuation^21,22^. We also suggest that given the significant interaction with PPIs resulting in a reduction in SVR, clinicians should address how best to control patient symptoms during DAA treatment^18,26^. In contrast to the results of these studies, we did not find a significant association of HCV genotype (e.g. genotype 3 or 1a) with increased risk of DAA treatment failure. The lack of this association may be due to the more common use now of pangenotypic DAA regimens for treating HCV.

Among the top ten predictors of DAA treatment failure, we identified several potentially modifiable risk factors, including tobacco, alcohol, and vitamin use. Several studies using National Health and Nutrition Examination Survey data have reported that individuals with HCV are nearly 3 times as likely to smoke tobacco (62% vs. 23%) and 3 times as likely to consume an average of more than 1 drink per day (36% vs. 14%) than individuals without HCV^31,32^. More than half of patients with HCV included in the present study smoked cigarettes, and 30% reported alcohol use at baseline, which is well representative of patients with HCV in the U.S. Another study found that 32% of patients with HCV treatment reported alcohol use and that unhealthy alcohol users (adjusted odds ratio, 0.75) among veterans with HCV were less likely to achieve SVR than those who were abstinent or reported low-level alcohol use^33^. Although the present study results suggested that the vast majority of patients with HCV were cured in the era of available DAA therapy, tobacco and alcohol use may be associated with decreased SVR rate or may serve as markers for patients at risk of treatment failure. It is also important to recognize that continued alcohol and tobacco use even after SVR may put patients at risk for progression of liver disease^31,33^.

The use of vitamins as a factor associated with DAA treatment failure has not been previously reported, but several studies have demonstrated that deficiency in vitamin A or vitamin D is a risk factor for fibrosis and for interferon/ribavirin treatment failure among patients with HCV infection^34–36^. We postulate that it may not be the use of vitamins themselves that is associated with DAA treatment failure, but that their use may be a proxy measure for the presence of a vitamin deficiency or malnutrition. A recent meta-analysis reported that low vitamin levels are common among patients with HCV infection and are associated with advanced liver fibrosis^37,38^. In the present study, we also found that patients with vitamin use were more likely to be older (59.7 vs. 56.4 years) and have advanced liver diseases (e.g. cirrhosis 39.5% vs. 34.0%; hepatocellular carcinoma 8.7% vs. 4.3%) [data not shown]. Nonetheless, further research is needed to validate our findings and to determine why multivitamin use may be adversely associated with or interact with DAAs.

Together, the findings of the present study have important implications for clinicians. First, in the high-risk group with many known risk factors, more than 95% of the group achieved SVR, suggesting that despite these risk factors, DAA treatment should be prescribed when indicated. Second, pretreatment risk factors, such as indictors of liver function, while important should not be used as discriminators when making decisions regarding whether to prescribe treatment. Rather, clinicians should continuously encourage treatment adherence and offer alternative ways to address modifiable risk factors (e.g. smoking cessation).

This study has several limitations. First, we did not include data on baseline resistance-associated substitutions (RASs) in NS3 or NS5A proteins. Although RASs have been associated with treatment failure, their contribution appears to be minimal and routine baseline RAS testing is not recommended^39,40^. Second, although we did not use an external validation data set, the present study expanding on the recent study by members of our group^18^ found that the factors associated with SVR failure remained similar to those of the previous study. In addition, we used data from the HCV-TARGET registry, one of the largest databases for patients with HCV infection prescribed DAAs in the U.S. Nevertheless, our results should be validated with other data sets. Third, machine learning requires extensive amounts of data and may incur high computational costs. However, by selecting the most parsimonious model (the EN model with 25 predictors), we reduced computational costs while maintaining high prediction validity and accuracy. Fourth, it is important that our results not be interpreted as causal inference between individual predictors and treatment failure but as associations.

In conclusion, the present study demonstrated that machine learning approaches provide a simple algorithm for identifying factors predictive of DAA treatment failure. Given that the number of HCV infection cases is increasing and that the number of patients with the infection still requiring treatment is high, addressing modifiable factors of DAA treatment failure (tobacco, alcohol, vitamin and PPI use) may reduce the burden of retreatment. Machine learning algorithms have the potential to inform public health policies regarding curative treatment of HCV.

## Supplementary Information

Supplementary Information.

## Data Availability

The data that support the findings of this study are available from HCV-TARGET research consortium, but restrictions apply to the availability of these data, which were used under license for the current study, and so are not publicly available. Data are however available from the authors (Joy Peter; joy.peter@medicine.ufl.edu) upon reasonable request and with permission of HACV-TARGET research consortium.

## Abbreviations

DAA: Direct-acting antiviral
EN: Elastic net
HCV: Hepatitis C virus
NNE: Number needed to evaluate
PPI: Proton pump inhibitor
SVR: Sustained virologic response
