# Supplementary material for: Evaluation of machine learning algorithms for predicting direct-acting antiviral treatment failure among patients with chronic hepatitis C infection
Source: Sci Rep. 2022 Oct 27;12:18094. doi: 10.1038/s41598-022-22819-4 (PMC9613877; doi:10.1038/s41598-022-22819-4)
Supplement: Supplementary file 1 — Supplementary Information. [file 41598_2022_22819_MOESM1_ESM.docx]

**Supplemental Table 1. Compliance with the 2015 Transparent Reporting of a Multivariable Prediction Model for Individual Prognosis Or Diagnosis (TRIPOD) Checklist**

| **Section/Topic** | **Item** |  | **Checklist Item** | **Page** |
| --- | --- | --- | --- | --- |
| **Title and abstract** | | | | |
| Title | 1 | D;V | Identify the study as developing and/or validating a multivariable prediction model, the target population, and the outcome to be predicted. | 1 |
| Abstract | 2 | D;V | Provide a summary of objectives, study design, setting, participants, sample size, predictors, outcome, statistical analysis, results, and conclusions. | 3 |
| **Introduction** | | | | |
| Background and objectives | 3a | D;V | Explain the medical context (including whether diagnostic or prognostic) and rationale for developing or validating the multivariable prediction model, including references to existing models. | 4 |
|  | 3b | D;V | Specify the objectives, including whether the study describes the development or validation of the model or both. | 5 |
| **Methods** | | | | |
| Source of data | 4a | D;V | Describe the study design or source of data (e.g., randomized trial, cohort, or registry data), separately for the development and validation data sets, if applicable. | 5 |
|  | 4b | D;V | Specify the key study dates, including start of accrual; end of accrual; and, if applicable, end of follow-up. | 5 |
| Participants | 5a | D;V | Specify key elements of the study setting (e.g., primary care, secondary care, general population) including number and location of centres. | 5 |
|  | 5b | D;V | Describe eligibility criteria for participants. | 5 |
|  | 5c | D;V | Give details of treatments received, if relevant. | 5 |
| Outcome | 6a | D;V | Clearly define the outcome that is predicted by the prediction model, including how and when assessed. | 6 |
|  | 6b | D;V | Report any actions to blind assessment of the outcome to be predicted. | NA |
| Predictors | 7a | D;V | Clearly define all predictors used in developing or validating the multivariable prediction model, including how and when they were measured. | 6-7; Supplemental Table 2; Supplemental Methods |
|  | 7b | D;V | Report any actions to blind assessment of predictors for the outcome and other predictors. | NA |
| Sample size | 8 | D;V | Explain how the study size was arrived at. | 8 |
| Missing data | 9 | D;V | Describe how missing data were handled (e.g., complete-case analysis, single imputation, multiple imputation) with details of any imputation method. | Supplemental Methods |
| Statistical analysis methods | 10a | D | Describe how predictors were handled in the analyses. | 6-7; Supplemental Methods; |
|  | 10b | D | Specify type of model, all model-building procedures (including any predictor selection), and method for internal validation. | 6-7; Appendix Methods |
|  | 10c | V | For validation, describe how the predictions were calculated. | 6-7; Supplemental Figure 1 |
|  | 10d | D;V | Specify all measures used to assess model performance and, if relevant, to compare multiple models. | 8-9; Supplemental Figure 1 |
|  | 10e | V | Describe any model updating (e.g., recalibration) arising from the validation, if done. | 9; Appendix Methods |
| Risk groups | 11 | D;V | Provide details on how risk groups were created, if done. | 9; Appendix Methods |
| Development vs. validation | 12 | V | For validation, identify any differences from the development data in setting, eligibility criteria, outcome, and predictors. | 9 |

| **Results** | | | | |
| --- | --- | --- | --- | --- |
| Participants | 13a | D;V | Describe the flow of participants through the study, including the number of participants with and without the outcome and, if applicable, a summary of the follow-up time. A diagram may be helpful. | 8 |
|  | 13b | D;V | Describe the characteristics of the participants (basic demographics, clinical features, available predictors), including the number of participants with missing data for predictors and outcome. | 8; Table 1; |
|  | 13c | V | For validation, show a comparison with the development data of the distribution of important variables (demographics, predictors and outcome). | will provide on request owing to a large amount of results |
| Model development | 14a | D | Specify the number of participants and outcome events in each analysis. | Tables 1 & 2 |
|  | 14b | D | If done, report the unadjusted association between each candidate predictor and outcome. | Consult investigators will provide on request owing to large tables |
| Model specification | 15a | D | Present the full prediction model to allow predictions for individuals (i.e., all regression coefficients, and model intercept or baseline survival at a given time point). | Consult investigators |
|  | 15b | D | Explain how to the use the prediction model. | Supplemental Methods |
| Model performance | 16 | D;V | Report performance measures (with CIs) for the prediction model. | Figures 1-2 |
| Model-updating | 17 | V | If done, report the results from any model updating (i.e., model specification, model performance). | Figures 1-2 |
| **Discussion** | | | | |
| Limitations | 18 | D;V | Discuss any limitations of the study (such as nonrepresentative sample, few events per predictor, missing data). | 12-13 |
| Interpretation | 19a | V | For validation, discuss the results with reference to performance in the development data, and any other validation data. | 12-13 |
|  | 19b | D;V | Give an overall interpretation of the results, considering objectives, limitations, results from similar studies, and other relevant evidence. | 10-13 |
| Implications | 20 | D;V | Discuss the potential clinical use of the model and implications for future research. | 10-13 |
| **Other information** | | | | |
| Supplementary information | 21 | D;V | Provide information about the availability of supplementary resources, such as study protocol, Web calculator, and data sets. | Online supplement |
| Funding | 22 | D;V | Give the source of funding and the role of the funders for the present study. | 2 |

*Items relevant only to the development of a prediction model are denoted by D, items relating solely to a validation of a prediction model are denoted by V, and items relating to both are denoted D;V. We recommend using the TRIPOD Checklist in conjunction with the TRIPOD Explanation and Elaboration document. **NA denotes not applicable.**

**Supplemental Table 2. Summary of predictor candidates (n=359) measured before and after DAA initiation**

|  | **Sociodemographic characteristics** | **Clinical factors** | **Laboratory data*** | **DAA treatment and adverse events** |
| --- | --- | --- | --- | --- |
| Before DAA treatment  (n=242) | - Sex - Age - Race - Ethnicity | - Liver condition - Comorbidities - Drug use - Tobacco - Alcohol - PPI dose and frequency - Immunosuppressant dose and frequency | - Genotype - HCV RNA - Albumin - Total bilirubin - Neutrophils - Alanine aminotransferase - Aspartate aminotransferase - Alkaline phosphatase - Neutrophil count - Glucose - Hemoglobin - Leukocytes - Lymphocytes - Platelets - Potassium - Sodium - Creatinine - Estimated glomerular filtration - Aspartate Aminotransferase to platelet ratio index - International normalized ratio - MELD score | - Treatment status (naïve, experienced, DAA experienced) - DAA regimen |
| On-treatment (n=117) | - None | - None | - HCV RNA - Albumin - Total bilirubin - Neutrophils - Alanine aminotransferase - Aspartate aminotransferase - Alkaline phosphatase - Neutrophil count - Glucose - Hemoglobin - Leukocytes - Lymphocytes - Platelets - Potassium - Sodium - Creatinine - MELD score | - Treatment duration - Treatment discontinuation due to adverse events - Adverse events (i.e., anemia, pruritus, rash, fatigue, dyspnea, insomnia, influenza like illness, irritability, nausea, cough, diarrhea, dizziness, decreased appetite, infection, palpitations, abnormal pain, tachycardia, hyperglycemia, renal failure, gastrointestinal hemorrhage, pyrexia, angina, bradycardia, arthralgia, gout, arrhythmia, pancytopenia, dehydration) |

Abbreviations: DAA, direct-acting antiviral; HCV, hepatitis C virus; MELD, model for end-stage liver disease; PPI, proton pump inhibitor.

*Laboratory data were measured before DAA treatment and week 4 visit.

**Supplemental Table 3. Performance measures for predicting DAA treatment failure across different machine learning methods with varying sensitivity and specificity.**

| Method | Score threshold (range 0-100)^a^ | Predicted treatment failure (%) | Sensitivity (%) | Specificity (%) | PPV (%) | NPV (%) | F1 score (%) | PLR | NNE |  |
| --- | --- | --- | --- | --- | --- | --- | --- | --- | --- | --- |
| **Elastic Net** | | | | | | | | | | |
| **Sensitivity** |  |  |  |  |  |  |  |  |  |  |
| 100% | 24.25 | 99.02 | 100.00 | 1.03 | 4.77 | 100.00 | 0.0910 | 1.01 | 21 |  |
| 99% | 28.20 | 93.56 | 98.70 | 6.69 | 4.98 | 99.05 | 0.0948 | 1.06 | 20 |  |
| 98% | 30.69 | 87.31 | 97.40 | 13.19 | 5.27 | 99.03 | 0.0999 | 1.12 | 19 |  |
| 97% | 30.69 | 87.31 | 97.40 | 13.19 | 5.27 | 99.03 | 0.0999 | 1.12 | 19 |  |
| 96% | 32.02 | 83.75 | 96.10 | 16.86 | 5.42 | 98.87 | 0.1026 | 1.16 | 18 |  |
| 95% | 34.84 | 76.03 | 94.81 | 24.90 | 5.89 | 98.98 | 0.1109 | 1.26 | 17 |  |
| 94% | 35.26 | 74.25 | 93.51 | 26.71 | 5.95 | 98.81 | 0.1118 | 1.28 | 17 |  |
| 93% | 35.26 | 74.25 | 93.51 | 26.71 | 5.95 | 98.81 | 0.1118 | 1.28 | 17 |  |
| 92% | 36.33 | 71.86 | 92.21 | 29.15 | 6.06 | 98.69 | 0.1137 | 1.30 | 17 |  |
| 91% | 39.36 | 59.66 | 90.91 | 41.89 | 7.19 | 98.94 | 0.1333 | 1.56 | 14 |  |
| 90% | 40.39 | 57.33 | 89.61 | 44.27 | 7.38 | 98.85 | 0.1364 | 1.61 | 14 |  |
| **Optimized threshold^b^** | 52.53 | 23.91 | 58.44 | 77.80 | 11.54 | 97.42 | 0.1927 | 2.63 | 9 |  |
| **Specificity** |  |  |  |  |  |  |  |  |  |  |
| 90% | 61.89 | 11.22 | 35.06 | 89.96 | 14.75 | 96.55 | 0.2077 | 3.49 | 7 |  |
| 91% | 63.40 | 10.18 | 31.17 | 90.86 | 14.46 | 96.38 | 0.1975 | 3.41 | 7 |  |
| 92% | 64.88 | 9.32 | 28.57 | 91.63 | 14.47 | 96.28 | 0.1921 | 3.42 | 7 |  |
| 93% | 65.91 | 8.28 | 25.97 | 92.60 | 14.81 | 96.19 | 0.1887 | 3.51 | 7 |  |
| 94% | 67.00 | 7.30 | 24.68 | 93.56 | 15.97 | 96.16 | 0.1939 | 3.83 | 6 |  |
| 95% | 69.37 | 5.70 | 23.38 | 95.17 | 19.35 | 96.16 | 0.2118 | 4.84 | 5 |  |
| 96% | 71.13 | 4.66 | 19.48 | 96.07 | 19.74 | 96.01 | 0.1961 | 4.96 | 5 |  |
| 97% | 72.98 | 3.49 | 14.29 | 97.04 | 19.30 | 95.81 | 0.1642 | 4.83 | 5 |  |
| 98% | 74.83 | 2.39 | 9.09 | 97.94 | 17.95 | 95.60 | 0.1207 | 4.41 | 6 |  |
| 99% | 78.33 | 1.41 | 6.49 | 98.84 | 21.74 | 95.52 | 0.1000 | 5.61 | 5 |  |
| 100% | 91.15 | 0.06 | 1.30 | 100.00 | 100.00 | 95.34 | 0.0256 | INF | 1 |  |
| **Maximized PPV** | 91.15 | 0.06 | 1.30 | 100.00 | 100.00 | 95.34 | 0.0256 | INF | 1 |  |
| **Method** | **Score threshold (range 0-100)** | **Predicted treatment failure (%)** | **Sensitivity (%)** | **Specificity (%)** | **PPV (%)** | **NPV (%)** | **F1 score (%)** | **PLR** | **NNE** |  |
| **FNN** | | | | | | | | | | |
| **Sensitivity** |  |  |  |  |  |  |  |  |  |  |
| 100% | 23.78 | 97.49 | 100.00 | 2.64 | 4.84 | 100.00 | 0.0924 | 1.03 | 21 |  |
| 99% | 28.36 | 88.41 | 98.70 | 12.10 | 5.27 | 99.47 | 0.1001 | 1.12 | 19 |  |
| 98% | 29.63 | 85.90 | 97.40 | 14.67 | 5.35 | 99.13 | 0.1015 | 1.14 | 19 |  |
| 97% | 29.63 | 85.90 | 97.40 | 14.67 | 5.35 | 99.13 | 0.1015 | 1.14 | 19 |  |
| 96% | 29.67 | 85.59 | 96.10 | 14.93 | 5.30 | 98.72 | 0.1005 | 1.13 | 19 |  |
| 95% | 31.71 | 80.93 | 94.81 | 19.76 | 5.53 | 98.71 | 0.1045 | 1.18 | 18 |  |
| 94% | 32.02 | 79.83 | 93.51 | 20.85 | 5.53 | 98.48 | 0.1044 | 1.18 | 18 |  |
| 93% | 32.02 | 79.83 | 93.51 | 20.85 | 5.53 | 98.48 | 0.1044 | 1.18 | 18 |  |
| 92% | 33.99 | 73.88 | 92.21 | 27.03 | 5.89 | 98.59 | 0.1108 | 1.26 | 17 |  |
| 91% | 36.00 | 67.75 | 90.91 | 33.40 | 6.33 | 98.67 | 0.1184 | 1.36 | 16 |  |
| 90% | 36.55 | 66.09 | 89.61 | 35.07 | 6.40 | 98.55 | 0.1195 | 1.38 | 16 |  |
| Optimized threshold | 51.05 | 24.77 | 63.64 | 77.16 | 12.13 | 97.72 | 0.2037 | 2.79 | 8 |  |
| **Specificity** |  |  |  |  |  |  |  |  |  |  |
| 90% | 60.51 | 11.22 | 33.77 | 89.90 | 14.21 | 96.48 | 0.2000 | 3.34 | 7 |  |
| 91% | 61.41 | 10.36 | 31.17 | 90.67 | 14.20 | 96.37 | 0.1951 | 3.34 | 7 |  |
| 92% | 63.42 | 8.71 | 29.87 | 92.34 | 16.20 | 96.37 | 0.2100 | 3.90 | 6 |  |
| 93% | 64.41 | 7.97 | 28.57 | 93.05 | 16.92 | 96.34 | 0.2126 | 4.11 | 6 |  |
| 94% | 65.54 | 6.93 | 25.97 | 94.02 | 17.70 | 96.25 | 0.2105 | 4.34 | 6 |  |
| 95% | 66.30 | 6.19 | 23.38 | 94.66 | 17.82 | 96.14 | 0.2022 | 4.38 | 6 |  |
| 96% | 68.33 | 4.78 | 18.18 | 95.88 | 17.95 | 95.94 | 0.1806 | 4.41 | 6 |  |
| 97% | 69.87 | 3.74 | 16.88 | 96.91 | 21.31 | 95.92 | 0.1884 | 5.47 | 5 |  |
| 98% | 71.65 | 2.76 | 14.29 | 97.81 | 24.44 | 95.84 | 0.1803 | 6.53 | 4 |  |
| 99% | 75.28 | 1.41 | 7.79 | 98.91 | 26.09 | 95.58 | 0.1200 | 7.12 | 4 |  |
| 100% | 86.61 | 0.06 | 1.30 | 100.00 | 100.00 | 95.34 | 0.0256 | INF | 1 |  |
| **Maximized PPV** | **86.61** | **0.06** | **1.30** | **100.00** | **100.00** | **95.34** | **0.0256** | **INF** | **1** |  |
| **Method** | **Score threshold (range 0-100)** | **Predicted treatment failure (%)** | **Sensitivity (%)** | **Specificity (%)** | **PPV (%)** | **NPV (%)** | **F1 score (%)** | **PLR** | **NNE** |  |
| **RF** | | | | | | | | | | |
| **Sensitivity** |  |  |  |  |  |  |  |  |  |  |
| 100% | 33.22 | 90.13 | 100.00 | 10.36 | 5.24 | 100.00 | 0.0995 | 1.12 | 19 |  |
| 99% | 33.72 | 88.17 | 98.70 | 12.36 | 5.29 | 99.48 | 0.1003 | 1.13 | 19 |  |
| 98% | 34.75 | 82.83 | 97.40 | 17.89 | 5.55 | 99.29 | 0.1050 | 1.19 | 18 |  |
| 97% | 34.75 | 82.83 | 97.40 | 17.89 | 5.55 | 99.29 | 0.1050 | 1.19 | 18 |  |
| 96% | 34.99 | 81.79 | 96.10 | 18.92 | 5.55 | 98.99 | 0.1049 | 1.19 | 18 |  |
| 95% | 35.46 | 78.85 | 94.81 | 21.94 | 5.68 | 98.84 | 0.1071 | 1.21 | 18 |  |
| 94% | 35.95 | 76.15 | 93.51 | 24.71 | 5.80 | 98.71 | 0.1092 | 1.24 | 17 |  |
| 93% | 35.95 | 76.15 | 93.51 | 24.71 | 5.80 | 98.71 | 0.1092 | 1.24 | 17 |  |
| 92% | 36.53 | 72.84 | 92.21 | 28.12 | 5.98 | 98.65 | 0.1123 | 1.28 | 17 |  |
| 91% | 36.76 | 71.06 | 90.91 | 29.92 | 6.04 | 98.52 | 0.1133 | 1.30 | 17 |  |
| 90% | 37.02 | 69.16 | 89.61 | 31.85 | 6.12 | 98.41 | 0.1145 | 1.31 | 16 |  |
| Optimized threshold | 41.85 | 35.07 | 75.32 | 66.92 | 10.14 | 98.21 | 0.1787 | 2.28 | 10 |  |
| **Specificity** |  |  |  |  |  |  |  |  |  |  |
| 90% | 49.10 | 11.04 | 32.47 | 90.03 | 13.89 | 96.42 | 0.1946 | 3.26 | 7 |  |
| 91% | 49.67 | 10.30 | 29.87 | 90.67 | 13.69 | 96.31 | 0.1878 | 3.20 | 7 |  |
| 92% | 51.16 | 8.52 | 28.57 | 92.47 | 15.83 | 96.31 | 0.2037 | 3.79 | 6 |  |
| 93% | 51.16 | 8.52 | 28.57 | 92.47 | 15.83 | 96.31 | 0.2037 | 3.79 | 6 |  |
| 94% | 53.06 | 6.62 | 24.68 | 94.27 | 17.59 | 96.19 | 0.2054 | 4.31 | 6 |  |
| 95% | 53.98 | 5.95 | 22.08 | 94.85 | 17.53 | 96.09 | 0.1954 | 4.29 | 6 |  |
| 96% | 55.53 | 4.72 | 18.18 | 95.95 | 18.18 | 95.95 | 0.1818 | 4.48 | 5 |  |
| 97% | 56.82 | 3.62 | 15.58 | 96.98 | 20.34 | 95.87 | 0.1765 | 5.15 | 5 |  |
| 98% | 58.22 | 2.94 | 14.29 | 97.62 | 22.92 | 95.83 | 0.1760 | 6.00 | 4 |  |
| 99% | 61.08 | 1.53 | 7.79 | 98.78 | 24.00 | 95.58 | 0.1176 | 6.37 | 4 |  |
| 100% | 66.81 | 0.06 | 1.30 | 100.00 | 100.00 | 95.34 | 0.0256 | INF | 1 |  |
| Maximized PPV | 66.81 | 0.06 | 1.30 | 100.00 | 100.00 | 95.34 | 0.0256 | INF | 1 |  |

| **Method** | **Score threshold (range 0-100)** | **Predicted treatment failure (%)** | **Sensitivity (%)** | **Specificity (%)** | **PPV (%)** | **NPV (%)** | **F1 score (%)** | **PLR** | **NNE** |
| --- | --- | --- | --- | --- | --- | --- | --- | --- | --- |
| **GBM** | | | | | | | | | |
| **Sensitivity** |  |  |  |  |  |  |  |  |  |
| 100% | 208.64 | 95.59 | 100.00 | 4.63 | 4.94 | 100.00 | 0.0941 | 1.05 | 20 |
| 99% | 213.86 | 93.50 | 98.70 | 6.76 | 4.98 | 99.06 | 0.0949 | 1.06 | 20 |
| 98% | 229.33 | 88.29 | 97.40 | 12.16 | 5.21 | 98.95 | 0.0989 | 1.11 | 19 |
| 97% | 229.33 | 88.29 | 97.40 | 12.16 | 5.21 | 98.95 | 0.0989 | 1.11 | 19 |
| 96% | 230.76 | 87.80 | 96.10 | 12.61 | 5.17 | 98.49 | 0.0981 | 1.10 | 19 |
| 95% | 241.91 | 82.10 | 94.81 | 18.53 | 5.45 | 98.63 | 0.1031 | 1.16 | 18 |
| 94% | 245.25 | 80.01 | 93.51 | 20.66 | 5.52 | 98.47 | 0.1042 | 1.18 | 18 |
| 93% | 245.25 | 80.01 | 93.51 | 20.66 | 5.52 | 98.47 | 0.1042 | 1.18 | 18 |
| 92% | 247.73 | 78.72 | 92.21 | 21.94 | 5.53 | 98.27 | 0.1043 | 1.18 | 18 |
| 91% | 252.62 | 75.29 | 90.91 | 25.48 | 5.70 | 98.26 | 0.1073 | 1.22 | 18 |
| 90% | 256.38 | 72.23 | 89.61 | 28.64 | 5.86 | 98.23 | 0.1100 | 1.26 | 17 |
| Optimized threshold | 335.49 | 25.63 | 58.44 | 76.00 | 10.77 | 97.36 | 0.1818 | 2.43 | 9 |
| **Specificity** |  |  |  |  |  |  |  |  |  |
| 90% | 384.08 | 11.22 | 31.17 | 89.77 | 13.11 | 96.34 | 0.1846 | 3.05 | 8 |
| 91% | 389.53 | 10.24 | 29.87 | 90.73 | 13.77 | 96.31 | 0.1885 | 3.22 | 7 |
| 92% | 396.19 | 9.14 | 28.57 | 91.83 | 14.77 | 96.29 | 0.1947 | 3.50 | 7 |
| 93% | 414.40 | 7.36 | 27.27 | 93.63 | 17.50 | 96.29 | 0.2132 | 4.28 | 6 |
| 94% | 414.40 | 7.36 | 27.27 | 93.63 | 17.50 | 96.29 | 0.2132 | 4.28 | 6 |
| 95% | 423.13 | 5.95 | 24.68 | 94.98 | 19.59 | 96.22 | 0.2184 | 4.92 | 5 |
| 96% | 426.35 | 5.58 | 23.38 | 95.30 | 19.78 | 96.17 | 0.2143 | 4.98 | 5 |
| 97% | 438.04 | 3.99 | 22.08 | 96.91 | 26.15 | 96.17 | 0.2394 | 7.15 | 4 |
| 98% | 448.29 | 3.00 | 19.48 | 97.81 | 30.61 | 96.08 | 0.2381 | 8.90 | 3 |
| 99% | 467.13 | 1.41 | 11.69 | 99.10 | 39.13 | 95.77 | 0.1800 | 12.97 | 3 |
| 100% | 532.06 | 0.06 | 0.00 | 99.94 | 0.00 | 95.28 | N/A | 0.00 | INF |
| Maximized PPV | 483.16 | 0.80 | 7.79 | 99.55 | 46.15 | 95.61 | 0.1333 | 17.30 | 2 |

Abbreviations: DAA, direct-acting antiviral; FNN, feedforward neural network; GBM, gradient boosting machine; INF, infinity; N/A, not able to calculate; NNE, number needed to evaluate; NPV, negative predictive value; PLR, positive likelihood ratio; PPV, positive predictive value; RF, random forest.
^a^ Scores were calculated by predicted probability multiplied by 100. Score threshold refers to the score used to classify or predict individuals with DAA treatment failure (i.e., ≥ the threshold) vs. DAA treatment success (i.e., <threshold)
^b^ Optimized threshold was calculated by the Youden Index to achieve balanced sensitivity and specificity.

**Supplemental methods**

**Introduction**

In this study, our primary goal was prediction and the secondary goal was risk stratification (i.e., to identify subgroups of patients at similar risk of the outcome). First, we randomly allocated two-thirds of patients to the training sample and the remaining one-third to the validation sample based on the clinical characteristic distribution. We developed and tested prediction algorithms for DAA failure using four machine learning approaches: elastic net (EN), random forest (RF), gradient boosting machine (GBM), and feedforward neural network (FNN). For each approach, we fit the trained/refined algorithms based on the training sample and then applied the final algorithm in the validation sample to evaluate prediction performance.

Our model reporting complies with the Transparent Reporting of Multivariable Prediction Model for Individual Prognosis or Diagnosis (TRIPOD) reporting guidelines.[1, 2] We calculated the C statistic (or area under the receiver operating characteristic [ROC] curve) from the validation sample to assess discrimination (i.e., the extent to which patients predicted as high risk exhibit higher DAA failure rates compared with those predicted as low risk). We examined any difference in C statistics across different approaches using the DeLong test.[3] For each probability cutoff point, DAA failure was predicted with calculated probabilities above the cutoff point, whereas non-failure was predicted with probabilities below the cutoff point. Based on their true and predicted DAA failure status, patients were assigned to one of four groups (i.e., true positive [TP], false positive [FP], true negative [TN], or false negative [FN]) shown in the classification matrix (**Supplemental Figure 1**). Given that DAA failure events are rare outcomes and that C statistics do not incorporate information about the prevalence of the outcome, we reported other more appropriate metrics, including sensitivity, specificity, positive predictive value (PPV), negative predictive value (NPV), positive likelihood ratio (PLR), negative likelihood ratio (NLR), number needed to evaluate (NNE) to identify one DAA failure, and estimated rate of alerts to assess pre-implementation evaluation of our prediction algorithms (**Supplemental Figure 1**).[4] The optimal algorithm for a screening test depends on pre-test probability of the outcome, the values of TPs and TNs, and the costs of FP and FN. Because these factors vary from setting to setting (and some of them are subjective choices), no single cutoff point is suitable for every purpose. To compare performance across methods, we presented and assessed these prediction metrics (e.g., NNE) at the optimized threshold of the predicted probability that balances sensitivity and specificity as identified by the Youden index,[5] as well as at multiple levels of sensitivity and specificity (i.e., 90%-100%) to allow for risk-benefit evaluations of interventions triggered by positive tests using different thresholds defining high risk.

Second, based on the individual’s predicted probability of a DAA failure event, we stratified patients in the validation sample by decile risk subgroups, with the highest decile further split into three additional strata based on the top 1^st^, 2^nd^ to 5^th^, and 6^th^ to 10^th^ percentiles to enable closer examination of patients at highest risk of DAA failure. We evaluated calibration plots (the extent to which the predicted DAA failure risk agreed with the observed risks) by the risk subgroup. We briefly summarize our machine learning approaches in the next three sections.

**Regularized logistic regression: elastic net (EN) [6, 7]**

We chose to use EN regularization because it minimizes overfitting through parameter shrinkage and variable selection to create a parsimonious algorithm. It has been shown that an EN outperforms and is more efficient than traditional least absolute shrinkage and selection operator (LASSO) regularization.[8] Briefly, after forming a prediction model with logistic regression using all candidate variables, beta coefficients were penalized and lowered to deal with model overfitting. The magnitude of penalization was subsequently changed to create various models with different prediction errors in a cross-validation process, so the final model achieved optimal penalization based on the lowest prediction error. In regularized regression, variable selection was performed automatically by shrinking regression coefficients of some variables to zero. Specifically, the loss function in the EN regularization included log likelihood and regularization parameters λ_1_ and λ_2_ (smaller value indicating less penalization). The EN procedure generated a total of 40 candidate values for λ_1_ and λ_2_ from the training set. We used 5-fold cross-validation and the 1-standard error (1-SE) rule to select the optimal λ_1_ and λ_2_ in the final model in the training and testing samples. We standardized continuous variables to improve optimization and convergence of the models. Similar to traditional statistical methods (e.g., logistic regression), regularized regression methods cannot handle missing values, and they delete rows with missing data. For variables with missing information, we imputed with the median for continuous variables and with the most frequent category for categorical variables. Our candidate model contained all predictor candidates, quadratic transformation of non-normal distributed continuous variables, and two-way interaction between two predictors. Inclusion of quadratic transformations is commonly practiced in conducting EN regularization to accommodate potential non-linear relationships between key candidate predictors and outcomes. Regularized regression is expected to be more effective when (1) there are many more columns (predictors) than rows (observations), (2) the predictors available may be extremely highly correlated with each other, or (3) the goal is to find the most compact model yielding an acceptable performance. We used the sklearn package in Python 3.6 to perform the EN. All other parameters were set as default values. For EN regularized regression, individuals in the validation sample were assigned to one of the two predictive categories (i.e., DAA failure vs. non-failure) if the probability threshold was >0.51 (optimized threshold identified using the Youden Index), >0.8 (using the top 1 percentile of predicted scores in the training sample), >0.7 (using the top 5 percentile of the predicted scores), and >0.64 (using the top 10 percentile of the predicted scores).

**Tree-structured approaches: random forest (RF) [8, 9] and gradient boosting machine (GBM; stochastic gradient boosting) [10, 11]**

This study used two tree ensemble approaches: RF and GBM. An RF consists of a collection of trees grown in parallel, whereas a GBM consists of a series of trees grown in a sequential order of successive trees to minimize residual error. For the RF, at each split in a tree, a random sample of predictors is chosen. We followed the steps and rationale from the implementation of the RF framework presented by Chirkov et al. [12] Prior to conducting a RF, for variables with missing information, we imputed with the median for continuous variables and with the most frequent category for categorical variables. We used the “RandomForestClassifier*”* in the sklearn package of Python 3.6 for this study. The final fine-tuning parameters included the number of trees to build as 600 with maximum depth as 5, the number of predictor candidates randomly selected at each node as $\sqrt{number of total predictors}=\sqrt{359}$, using the balanced (i.e., upweight small classes to equal the size of the largest target class) class weight function. For the RF, validation visits were assigned to one of the two predictive categories (i.e., DAA failure vs. non-failure) if the probability threshold was >0.46, which was identified from the ROC curve using the Youden Index.

For the GBM, we used the “XGBClassifier” model in the xgboost package of Python 3.6. Using cross entropy (i.e., negative average log likelihood) as the tuning criterion, we optimized the hyper parameters of the classifier by 5-fold cross-validation and determined the final GBM model by fitting the training samples with 200 trees, maximum depth as 2, minimum child weight sum as 1, maximum delta step in prediction value as 1, minimum loss reduction required to make further partition as 0, and a learning rate that applies to each update as 0.3. In each tree, the splitting predictor at each level was optimized over 70% of the predictors selected randomly with 70% of the records sampled randomly in the leaf. Finally, we validated the algorithms with validation samples by assigning each patient to one of the two predictive categories (i.e., DAA failure vs. non-failure) based on a probability threshold of 0.49 that was identified from the ROC curve using the Youden Index.

**Feedforward neural network (FNN) [13, 14]**

We used a feedforward neural network (FNN) to develop algorithms in our training data sets using Python 3.6 (*keras* package). We examined different numbers of hidden layers and nodes. At the end, the FNN with 2 hidden layers with 359 nodes (i.e., the number of predictors we used) performed the best. In each hidden layer during the algorithm-training process, we chose ReLU as an activation function to better prevent a vanishing gradient and to yield a faster convergence. We applied a sigmoid function to the output layer to generate a score representing the probability of treatment success/failure. We used the binary cross-entropy loss function with balanced class weight to adjust for a rare outcome and used the C statistic as an early stopping criteria during cross-validation to prevent overfitting. Finally, we conducted a hyperparameter search using the grid L1 and L2 regularization weight, predictor dropout rate, and subsampling rate. For the FNN, validation visits were assigned to one of the two predictive categories (i.e., DAA failure vs. non-failure) based on a probability threshold of 0.48 identified from the ROC curve using the Youden Index.

**Supplemental References**

1. Collins GS, Reitsma JB, Altman DG, Moons KG. Transparent Reporting of a multivariable prediction model for Individual Prognosis or Diagnosis (TRIPOD): the TRIPOD statement. Ann Intern Med. 2015;162(1):55-63. Epub 2015/01/07. doi: 10.7326/M14-0697. PubMed PMID: 25560714.

2. Bossuyt PM, Reitsma JB, Bruns DE, Gatsonis CA, Glasziou PP, Irwig L, et al. STARD 2015: an updated list of essential items for reporting diagnostic accuracy studies. BMJ. 2015;351:h5527. Epub 2015/10/30. doi: 10.1136/bmj.h5527. PubMed PMID: 26511519; PubMed Central PMCID: PMCPMC4623764.

3. DeLong ER, DeLong DM, Clarke-Pearson DL. Comparing the areas under two or more correlated receiver operating characteristic curves: a nonparametric approach. Biometrics. 1988;44(3):837-45. Epub 1988/09/01. PubMed PMID: 3203132.

4. Romero-Brufau S, Huddleston JM, Escobar GJ, Liebow M. Why the C-statistic is not informative to evaluate early warning scores and what metrics to use. Crit Care. 2015;19:285. Epub 2015/08/14. doi: 10.1186/s13054-015-0999-1. PubMed PMID: 26268570; PubMed Central PMCID: PMCPMC4535737.

5. Fluss R, Faraggi D, Reiser B. Estimation of the Youden Index and its associated cutoff point. Biom J. 2005;47(4):458-72. Epub 2005/09/16. PubMed PMID: 16161804.

6. Lo-Ciganic WH, Huang JL, Zhang HH, Weiss JC, Wu Y, Kwoh CK, et al. Evaluation of Machine-Learning Algorithms for Predicting Opioid Overdose Risk Among Medicare Beneficiaries With Opioid Prescriptions. JAMA Netw Open. 2019;2(3):e190968. Epub 2019/03/23. doi: 10.1001/jamanetworkopen.2019.0968. PubMed PMID: 30901048.

6. Hastie T, Tibshirani R, Friedman J. The Elements of Statistical Learning: : Data Mining, Inference, and Prediction. 2nd ed. New York, NY: Springer; 2008.

7. Zou H, Hastie T. Regularization and variable selection via the elastic net. J R Stat Soc Series B Stat Methodol. 2005;67(Part 2):301-20.

8. Breiman L. Random Forest. Machine Learning. 2001;45:5-32.

9. Boulesteix AL, Janitza S, Kruppa J, Konig I. Overview of random forest methodology and practical guidance with emphasis on computational biology and bioinformatics. Wiley Interdisciplinary Reviews: Data Mining and Knowledge Discovery. 2012;2(6):493-507.

10. Friedman JH. Greedy Function Approximation: A Gradient Boosting Machine. Technical report, Dept. of Statistics, Stanford University. 1999.

11. Friedman JH. A Gradient Boosting Machine. Annals of statistics. 2001;29(5):1189.

12. Chirikov VV, Shaya FT, Onukwugha E, Mullins CD, dosReis S, Howell CD. Tree-based Claims Algorithm for Measuring Pretreatment Quality of Care in Medicare Disabled Hepatitis C Patients. Med Care. 2015. doi: 10.1097/MLR.0000000000000405. PubMed PMID: 26225448.

13. LeCun Y, Bengio Y, Hinton G. Deep learning. Nature. 2015;521(7553):436-44. Epub 2015/05/29. doi: 10.1038/nature14539. PubMed PMID: 26017442.

14. Schmidhuber J. Deep learning in neural networks: an overview. Neural Netw. 2015;61:85-117. Epub 2014/12/03. doi: 10.1016/j.neunet.2014.09.003. PubMed PMID: 25462637.

Supplemental Figure 1. **Classification matrix and definition of prediction performance metrics**

**
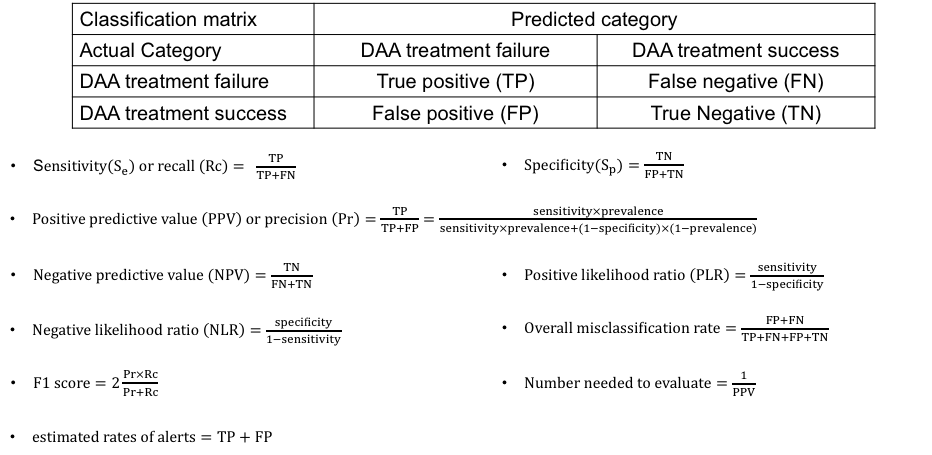
**

| **Prediction metrics** | **Definition** |
| --- | --- |
| Sensitivity (Se ) or recall (Rc) | The proportion of correctly predicted positive individuals with DAA treatment failure (i.e., predicted DAA treatment failure) divided by all individuals with actual DAA treatment failure. |
| Specificity (Sp) | The proportion of correctly predicted negative individuals (i.e., predicted DAA treatment success) divided by all observations with actual DAA treatment success. |
| Positive predictive value (PPV) or precision (Pr) | The proportion of actual DAA treatment failure cases divided by all individuals predicted as DAA treatment failure. PPV is influenced by the prevalence of the outcome of interest. |
| Negative predictive value (NPV) | The proportion of actual DAA treatment success cases divided by all observations predicted as DAA treatment success. When the outcome is rare, NPV is typically high. |
| Positive likelihood ratio (PLR) | The probability that a person with an actual incident DAA treatment failure is predicted as DAA treatment failure, divided by the probability of a person who did not have an actual incident DAA treatment failure is predicted as DAA treatment failure. The larger the PLR (>1), the better the prediction performance of an algorithm. |
| Negative likelihood ratio (NLR) | The probability that a person with an actual incident DAA treatment failure is predicted as DAA treatment success, divided by the probability that a person who did not have an actual DAA treatment failure is predicted as DAA treatment success. The smaller the NLR (i.e., closer to 0), the better the prediction performance. |
| Overall misclassification rate | The proportion of incorrectly predicted observations (i.e., false positives and false negatives of DAA treatment failure) divided by the total observations. |
| F1 score | The weighted average of precision (or PPV) and recall (or sensitivity). F1 takes both false positives and false negatives into account, and it is usually more useful than the overall misclassification rate under an uneven class distribution (e.g., DAA treatment success individuals comprised the majority of the cohort).[1] An F1 closer to 1 is desirable. |
| C statistic | The area under the receiver operating characteristic curve (ROC) curve, which is a plot of sensitivity vs. (1-specificity) for all potential cutoff probability thresholds for an algorithm. Comparisons of C statistics based on imbalanced data or rare outcomes can be misleading because C statistics do not incorporate information about prevalence or pre-test probability of the outcome.[2] |
| Precision-recall curves | A precision-recall curve of precision (or PPV; y-axis) vs. recall (sensitivity; x-axis). The curve closer to the upper right corner (corresponding to 100% precision and 100% recall) has better performance. |
| Number needed to evaluate (NNE) | The NNE is the number of patients necessary to evaluate or screen to detect one outcome (i.e., DAA treatment failure), similar to number needed to treat in clinical trials. A PPV of 10% is equivalent to an NNE of 10. |
| Estimated rate of alerts | Provides the estimated number of alerts per number of patients screened or evaluated over a period of time, for example, per 100 patients over 3 months. Too many alerts may lead to alert fatigue; too few alerts may lead to unfamiliarity with the clinical response. |
